# How Funerals Accomplish Family: Findings From a Mass-Observation Study

**DOI:** 10.1177/0030222818804646

**Published:** 2018-10-06

**Authors:** Tony Walter, Tara Bailey

**Affiliations:** 1 University of Bath, UK

**Keywords:** funerals, family, grief, Mass-Observation, feeling

## Abstract

The article analyses how potentially conflicting frames of grief and family operate in a number of English funerals. The data come from the 2010 Mass-Observation directive “Going to Funerals” which asked its panel of correspondents to write about the most recent funeral they had attended. In their writings, grief is displayed through conventional understandings of family. Drawing on Randall Collins, we show how the funeral stratifies mourners into family or nonfamily, a stratification accomplished—by family and nonfamily—through both outward display and inner feeling. The funerals described were more about a very traditional notion of family than about grief; family trumped grief, or at least provided the frame through which grief could be written about; and perceptions of “family” prompted emotions which in turn defined family. The funerals were portrayed as a distinct arena privileging family over the fluid and varied personal attachments highlighted in both the new sociology of personal life and in the concept of disenfranchised grief.

## Introduction

Contemporary Anglophone societies frame grief in terms of a unique personal connection to the deceased, yet hedged about by expectations that people will grieve certain family members more than others or more than friends, neighbors or colleagues. Only at the funeral are all relevant parties likely to find themselves physically in the same place, so it comprises a significant arena in which to explore this ambiguity. Funeral professionals often assert that the funeral is an occasion to express grief and to share sorrow, yet it is also an occasion typically controlled by close family who make the arrangements for the day, whatever their feelings or lack of feelings for the deceased. The funeral director contracts with just one person as client, typically a close family member. Critics of the mid-20th century American funeral denigrated the way it became an ostentatious display of family status (Bowman, 1959); more recent critics have observed how funerals can disenfranchise the grief of nonfamily (Doka, 2002); anecdotal observations by British mourners and funeral professionals, along with more systematic observation by researchers (Clark, 1982; Howarth, 1996), indicate that close family always sit in the front row; and soap operas portray funerals as dramatic family events. So there are reasons to think that British and American funerals may have as much to do with family as with personal feeling.

Curiously, this has not been a research focus since Bowman’s (1959) polemical critique of American funeral expenditure as ostentatious family status display. Recent British sociological research has looked at how families accomplish funerals (Holloway, Adamson, Argyrou, Draper, & Mariau, 2013) but not how funerals accomplish family; in other words, contemporary funeral research does not problematize “family.” Hochschild’s (1983) discussion of grief, family roles, and funerals analyses the inner work required when personal grief does not match cultural expectations, but she assumes that funerals should help people express grief, an assumption our findings question. These studies contrast markedly with anthropological studies of rites at or after the funeral in East Asia where scholars often see these rites—originating in ancestor veneration rather than Christian hope—as legitimating and sustaining family structures (Aveline-Dubach, 2012). Eastern funerals may, of course, be more about grief than studies portray; we ask here whether Western funerals are more about family than studies portray. This article’s investigation into how a number of English funerals accomplish family is therefore somewhat innovative.

## Theoretical Tools

### Ritual

Central to Durkheim’s (1915) famous argument that religion functions to symbolize and affirm the group was his analysis of Aboriginal funeral rites. This prompts questions for the analysis of contemporary funerals: If funeral rites affirm the group that has lost a member, is that group the family (and if so, what does family mean?), or is it the entire assembly of diverse groups (work, social, neighborhood, etc.) to which the deceased belonged and in which she or he may have been cared for, liked, disliked, or respected? Durkheim’s view (1915) that rituals, not least mourning rituals, symbolize the group drew on studies of simple Aboriginal societies bound together in an undifferentiated “mechanical” solidarity. In larger and more complex societies characterized by what Durkheim (1933) called “organic” solidarity, Randall Collins (2004) argues that ritual may function to *differentiate* one group from another, insiders from outsiders. This raises the possibility that the funeral may function to differentiate different groups of mourners, for example, family versus nonfamily; this forms a central question in our analysis.

### New Sociology of Family

What, though, is “family”? Looked at one way, family remains an institution, with traditional family structures powerfully embedded in policy, law, and politics, irrespective of how people actually live (Edwards, Ribbens McCarthy, & Gillies, 2012). Alternatively, however, the “new” sociology of the family sees family not in terms of structured kinship but as something constructed through each family’s unique practices, reflecting new and diverse forms of doing family. Morgan’s (1996) notion of “doing” family through “family practices” understands families as constructed by everyday practices rather than by structures of genealogical relationship. Family is what family does. Finch’s concept of “displaying family”—how individuals and groups indicate “family” to others—offers, we suggest, a lens through which to research families at funerals. Displaying family is accomplished “primarily through direct social interaction with those with whom one is establishing family relationships” (Finch, 2007, p. 74); others then reinforce (or, presumably, challenge) this “family-ness.” Such others include other family members, friends, colleagues and employers, and public agencies, all of whom have at their disposal more and less formal means of endorsing family relationships.

Smart’s (2007) approach to “personal life” places family within a bigger framework still, encompassing a wide range of relationships, and follows anthropologist Carsten (2004) in seeing kinship not as a structure but as negotiated relatedness; kinship is constantly done and redone in everyday interaction. This highlighting of the personal significance of relationships irrespective of traditional notions of family fits well with thanatology’s concept of disenfranchised grief which argues that intense grief can be felt for anyone to whom one felt a close connection, irrespective of formal family relationship. We therefore now turn from the sociology of family to the sociology of grief.

### Social History of Grief

In both society and the academy today, grief is expected to reflect one’s structured kin relationship to the deceased (e.g., spouses grieve more than do nephews or grandchildren), *or* one’s personal attachment to the deceased, whatever the formal relationship. Like family, grief can be seen in terms of formal structures of kinship, or as personally constructed and experienced. Unpacking the sociological and historical context can illuminate why this is so.

Lofland (1985) identifies social structural and demographic reasons why within Western modernity grief is often felt more intensely than in many other societies. First, the modern nuclear family concentrates threads of connectedness in a few intimate relationships which, because of the dramatic extension of longevity, develop over many decades of shared experience. Spousal and parent–child relationships may last 60 years, and sibling relationships for 80 years or more. Such attachments can be strong and enduring and grief accordingly severe (Blauner, 1966). Second, modern Western notions of the individual locate grief within the individual’s inner life. Third, improved living standards provide people with more time, leisure and privacy to explore their inner grief.

For these reasons, grief for a close relative (spouse, children, parents, and siblings) can often be intense. Within families, however, antagonisms can be as strong and long lasting as positive attachments; relatives may not have spoken to each other for decades, others may simply drift apart. In reconstituted families, who is considered “family” is open to negotiation. At the same time, longevity means that friendships (Allan, 1979) and work relationships can also be long lasting (Fowlkes, 1990). Together, these conditions elicit constructions of grief as a family phenomenon *or* as an individual phenomenon; this article addresses how these two constructions interpenetrate.

Lou Taylor’s (1983) feminist history of English mourning dress relates grief to power, patriarchy, and emancipation. For Victorian upper class ladies with the leisure to grieve, the prescribed mourning period reflected not actual attachment to the deceased but patriarchal structures; thus, a woman was required to mourn her husband’s father for longer than her own child. By the 1890s, a decade in which the suffragist movement was demanding women’s right to vote, upper class women were also claiming the right to grieve according to their own lights; rejecting patriarchal norms, they wished their mourning to reflect personal attachment to the deceased. Whether voting or grieving, these women no longer considered themselves appendages of their husbands. The 20th century went on to further celebrate the mourner’s freedom, framing grief as an intensely personal matter to be endured in private, rather than a public act open to others’ surveillance and potential censure (Gorer, 1965). Later in the century, attachment theory came to dominate grief psychology, suggesting that grief has less to do with formal relationship to the deceased than with personal attachment, in turn, reflecting attachment styles formed in infancy (Parkes, 2008). By the 21st century, advice leaflets typically tell bereaved readers that there are no rules in grief, no prescribed stages or timelines, and that grief is entirely personal to the individual.

Yet grief today is far from entirely escaping cultural norms that validate some relationships over others; thus, the death of a child is widely assumed to be harder than the death of an elderly parent; the death of a spouse harder than the death of a sibling; and the death of a sibling harder than that of a friend or colleague (Doka, 2002; Fowlkes, 1990). In focusing on the loss of spouses and children, bereavement research on both sides of the Atlantic over many decades has reproduced cultural expectations about who should be mourned (Parkes, 2008). But personal grief does not always match such culturally expected feeling rules (Hochschild, 1983, pp. 56–63). The tension between these norms and personal freedom is expressed in Doka’s concept of disenfranchised grief, widely used by clinicians in the United States to argue that any grief has the right to be “enfranchised” or taken seriously and that a person’s grief cannot be inferred from his or her formal relationship to the deceased.^1^

How this is manifested in the funeral may be illuminated by American sociologist Arlie Hochschild’s concepts of display rules and feeling rules. Is the funeral primarily an occasion for expressing, feeling, and displaying grief or for expressing, feeling, and displaying family? How are attenders expected to behave and to feel? What do they think they should do and feel? In Hochschild’s (1983) terminology, what display rules and feeling rules operate? Specifically, how are family and feelings displayed? What does this tell us about how both family and grief are done and displayed in contemporary societies (Finch, 2007; Morgan, 1996)? In analyzing empirical data on some early 21st century English funerals, this article furthers a hitherto somewhat undeveloped conversation between the sociology of emotions, specifically the sociology of grief, and the sociology of the family.

## Methods

Obtaining extensive qualitative data on how people experience public events is something for which Mass-Observation (M-O) is particularly well suited. M-O is a long-running, large-scale qualitative writing project based and archived at the University of Sussex in which “correspondents” (the project’s participants) respond to “directives” sent to them 3 times a year. These are open-ended questionnaires on “themes which cover both very personal issues and wider political and social issues and events” (Mass-Observation Project, 2011). Our data come from the 2010 M-O directive *Going to Funerals* which we commissioned and which asked correspondents to write about the most recent funeral they had attended—most likely, not one they had themselves arranged.

This wording intended to capture, across all the correspondents, a wide range of mourners, from closely to distantly attached to the deceased, and included family, friends, neighbors, colleagues, club and association members, nurses and police officers, and so forth. Specifically, it captures how the whole range of attenders, not just close family, write about “doing” emotions and family. Previous sociological funeral research has focused on funeral professionals (Caswell, 2011; Howarth, 1996; Pine, 1975) or very close family (Holloway et al., 2013; Szmigin & Canning, 2015), whereas we wished to access the entire funeral congregation. The only other study to use a comparable methodology is O’Rourke, Sptizberg, & Hannawa’s (2011) study which asked American respondents about the most recent funeral they had attended, in their case by means of an online survey. Asking about the most recent funeral attended also ensured that correspondents were not selecting particularly memorable, wonderful, or ghastly funerals—we wanted ordinary mourners to write about ordinary funerals. Whether our method reveals how funerals construct family and emotion, or simply how M-O correspondents’ writings construct family and emotion, is a question we consider after we have presented our findings. Either way, the findings tell us something about social constructions of funerals, families, and grief in contemporary England.

The full (two page) text of the directive may be found in Appendix A of Bailey (2012). Near the start, we made clear that “It’s your relationships to other people that we’re mostly interested in” and we asked specific questions such as whose funeral it was, who they went with, and who else was there, but we did not mention the word “family.” We did, however, specifically ask about feelings: “What sort of feelings did you have at the funeral? Did anything in particular arouse emotions for you?” “Did you express your emotions? If so, how? If not, was there a particular reason?” To elicit any particularly strong views of funerals, we asked a supplementary question about the best and worst funerals attended, and—as the methodology otherwise excludes funerals not attended—whether correspondents had ever decided against going to a funeral, and why.

The 500 or so active M-O correspondents are somewhat elderly, with 64% aged over 50 years compared with 34% of the British population (Mass-Observation Project, 2011) but possibly reflecting the mean age of funeral attenders. There are more females than male correspondents, more middle and lower middle class than working class, and few from minority ethnic backgrounds; correspondents come from all over Britain but not evenly so (Sheridan, 2002). In sum, correspondents are disproportionately White, middle-class women over 50 years living in southeast England who enjoy writing. (This article therefore refers to English, rather than British, funerals—“English” alluding to ethnicity as well as geography.) A total of 241 correspondents replied to the *Funerals* directive, a normal M-O response rate. As with any M-O research, no claims are made that the sample represents, in this case, funeral attenders throughout the United Kingdom; older, White middle-class Englishwomen surely have particular ways of doing both family and emotion and writing about them. Although, therefore, thin on material from working class and younger mourners, our sample size remains impressive compared with other qualitative funeral research, producing data that are both rich and extensive.

Most of the funerals written about had taken place within the previous 2 to 4 years, with some correspondents writing soon afterward. Apart from two Pagan funerals, all the funerals were Christian or nonreligious (e.g., humanist). Funerals with no one attending were by definition not described. Replies varied in length; some comprised just a short paragraph, most between two and six sides, some considerably longer.

Of the 241 replies, 161 were handwritten or typed on paper, held at the Archive and 80 were electronic (normal M-O response rates). Given our geographical distance from the Archive, we read the electronic replies first and then visited the Archive to add paper replies that offered new material; after analysis, we revisited the Archive to see if we could find any material contradicting the analysis (we could not). Because the project was interested in those mourners whom researchers had hitherto not focused on, namely, everyone other than the person responsible for arranging the funeral in question, material (from nine correspondents) about funerals which they had themselves arranged was excluded.^2^ Thirty very short replies that appeared uninformative were also excluded, as were reports of funerals held in other countries and reports by three correspondents (two clergy and one coffin bearer) who had attended their last funeral in a professional capacity. After exclusions, 173 replies (122 by women, 49 by men, and 2 of unstated gender) form the basis of our inductive thematic analysis (Braun & Clarke, 2006). Correspondents’ grammatical errors have not been corrected; identifying names have been altered, but M-O code numbers are given so that other scholars can check our data and interpretations.

### English Funerals

Because funeral rites, practices, and customs vary considerably between and within modern Western societies (Walter, 2005), a sketch of the English funeral is required before we proceed to the findings. The largely White Christian or Humanist funerals that correspondents wrote about differ both from minority religious funerals in England and from funerals in other countries. About 75% of English funerals are cremations and 25% are burials, this figure masking regional and urban or rural variations. Although in North America, the funeral service is preceded by a social gathering open to anyone to view the deceased in the funeral home, viewing at English funeral premises is private, and the funeral director’s client, usually a close family member, may gate-keep who is allowed to view (Harper, 2010). The main English social ritual therefore is not viewing, but—with cremations—a service or ceremony in the chapel or hall with which every British crematorium is furnished, and—with burials—a service in a church or chapel after which some, but often not all, mourners proceed to the graveside. When English people talk of “going to a funeral” they mean attending the funeral service in church, chapel, or crematorium. In 2010, most funerals were led by a church minister, though a rapidly increasing number are now led by a celebrant independent of any religious organization. Immediately after the cremation or burial, there is usually a social gathering, referred to here as the funeral tea, in a pub, hotel, community hall, private home, or other venue.

Mourners typically choose, first, whether to attend the main service in church or crematorium, and then (if attending) what to wear, where to sit, and whether to go on to the tea; also whether to send flowers (of what kind) or a donation (how much, to which charity). English social norms leave considerable scope for freedom in answering such questions, illustrated by the M-O replies; correspondents’ choices were therefore not entirely determined, and reveal how mourners think about, feel, and display both family and emotion. In this article, we focus on attending the funeral, attending the tea, and displaying emotion. How, in writing about these things, do correspondents “do” family and emotion? We look first at family and then at emotion.

## Findings

### Displaying Family

Although the directive did not specifically ask about family, it is striking how often the term “the family” appears in correspondents’ writing, many correspondents identifying themselves as belonging, or not belonging, to the deceased’s “family.” It is also striking how their writing assumes that conventional rather than fluid definitions of family or friendship structure the funeral—and very largely *should* structure it.

#### Attending the funeral

One important way that people display the deceased’s family is to attend the funeral. Much of the time, going to a “family funeral” is simply “doing” family, in that it is simply what one does since one is family (Morgan, 1996). This is not to say that “doing” family in this way is always unproblematic, for close family members could feel compelled to attend when they would rather not:

As my husband left the crematorium he said he wouldn’t have gone if he thought he could have got away with it and only went for his mum’s sake … To say you actually hate rather than dislike your own father is such a huge leap but that’s how he and some of his brothers felt about him. (M2486, female, 58)

Whether or not to attend the funeral can pose a dilemma for family members who disliked or did not feel close to the deceased. As Morgan (1996) notes, doing family need not be experienced positively, as can be seen here:

I could feel daggers of resentment coming from a lady standing close to me (I had a distinct sense that she didn’t want to be there but had been “delegated” by the family as the only available representative). (H1745, female, 59)

When correspondents reported criticism of family members for nonattendance, the criticism seems to be that the expected display of family-ness did not in fact occur:

My twin nephews (aged 31 at the time) did not attend their grandmother’s funeral which I think was also regarded as very wrong of them. (O3436, female, 56)

The cousins, two girls, were apparently “too busy” to come, which Graham’s mother was very unimpressed by. (P2957, female, 41)

In these examples, not only are the reported critics of the failure to attend members of the family but so too are the correspondents reporting the events. Generally only correspondents who were themselves relatives could actually tell whether or not everyone who “should” have attended a family funeral was in fact there and could assess the validity of the excuse provided (Finch & Mason, 1993). However, other mourners could also expect the family to display their relatedness to the deceased through attendance:

It was fairly evident there were no relatives—other than me present. I heard muttering that the brother wasn’t there. (M3476, female, 55)

Thus, both family members themselves and other mourners had expectations about relatedness being done and displayed by the family. At the same time, relatives who chose not to attend presumably prioritized personal feeling over displaying family unity.

It is not *only* family that mourners may expect to see displayed at a funeral:

All in all it was a “good” funeral—all her family + friends were there. (W3730, female, 43)

In the main, however, approval for attendance and disapproval of absence were directed at the deceased’s relatives, highlighting the importance at a funeral of displaying family (Heapy, 2011).

Despite what we know in other contexts about both family and grief being gendered (Martin & Doka, 2000), whether or not people attended and where they sat did not depend on gender. This contrasts with the Western fringes of Britain, where still sometimes only males accompany the body to its burial—this did not feature in any correspondent’s account. Likewise, no accounts of seating even remotely resembled a large Catholic funeral attended by one of the authors in the Belgian city of Antwerp in the late 1980s which arranged women on one side and men on the other, thus separating the deceased’s widow from their 10-year-old son. The differentiation displayed in the M-O accounts is not male or female, but family or nonfamily.

#### Attending the tea

Whether to join the motor cortege and (with burials rather than cremations) whether after the main funeral service to go on to attend the burial were also questions that some correspondents asked themselves and answered in terms of whether or not they perceived themselves as “family.” The directive asked specifically about attending the postfuneral tea, so a lot of correspondents wrote about this, many of them distinguishing between family and nonfamily. Notions of not wanting to intrude were common, perhaps reflecting the sample’s largely south of England middle-class character.

… although the family invited everyone in the church back for some refreshment afterwards, we didn’t want to intrude, so instead we went to a nearby pub and had a pint of beer. (P2957, female, 41)

I didn’t go to the wake partly from my own cowardice and partly because the family didn’t need another guest to worry about, thank for coming, comfort etc. (A3434, female, 45)

Correspondents did write about refraining from intruding in other contexts, including not attending the funeral of someone not known to them and sitting at the back of the congregation. However, intrusion is mentioned most frequently in connection with attending (or not attending) the tea. What does this tell us?

Even less has been written about funeral teas in Britain than about funerals themselves—but for perspectives from other cultures, see Cann (2017), Grainger (1998), Yoder (1986). It is not clear how often instructions are given to mourners about attending the tea, but where correspondents mention a message being relayed to them, it is always an invitation rather than a restriction. This can be contrasted with explicit instructions about flowers—such as “Family flowers only”—designed to separate family from nonfamily. As we can see from P2957 discussed earlier, even when mourners do feel like sharing a drink and each other’s company, an invitation may not be enough to override their opinion that attendance at the tea would be an intrusion.

From the standpoint of sharing sorrow and supporting the family, this may be unfortunate. O’Rourke et al. (2011) shows that the most valued part of American funerals is not formal ritual but informal social interaction between family and others who knew the deceased; this typically takes place at the prefuneral wake at which widespread attendance is expected. In Britain, it takes place at the postfuneral tea; many M-O correspondents considered, rightly or wrongly, their attendance at this would blur the family or nonfamily boundary and undermine support. This considerably reduces the opportunities for nonfamily to share stories of the deceased with family, arguably a significant part of mourning (Walter, 1996).

### Displaying a Loving Family

Displaying family simply by being there was not always enough. Correspondents emphasize it is important to display a “proper,” “normal,” or “good” family and to display shared norms about these (Pahl & Spencer, 2010); in Finch’s (2007) words, “to convey the message ‘this is my family *and it works*’” (emphasis added, p. 70). This imperative may be especially important when a family is available for scrutiny by others (James & Curtis, 2010) and also when participating in rites of passage which invoke family continuity and predictability. As funerals are now expected to be personalized (Holloway et al., 2013), there is potential conflict between displaying the ideal family lived *by* and the actual family man or woman lived *with* (Gillis, 1997). What kind of family, then, did correspondents consider should be displayed at funerals?

Although the literature suggests that family forms not fully recognized as conventional are likely to call most intensely for public displays of family (Almack, 2011; Gabb, 2011; Weeks, Heaphy, & Donovan, 2001), only one correspondent confirmed family-ness in this way. He referred to the funeral as “a gay one” and affirmed both standard family funeral practices (speaking at the funeral) and family practices (staying together for many years). More significant in correspondents’ writing about funereal displays of a “proper family” are expectations about the *quality of family relationships*. The following extract describes a mother’s attempt to require a particular kind of display from her daughter—or more accurately, insisting a disruptive display did *not* occur:

The other worst funeral was my maternal grandfather’s. He was 91 when he died in 2002 in a nursing home, but I had not spoken to him since 1985 because he had sexually abused me, my sister, and my mother since childhood … My mother and sister are still in various stages of denial about it … My mother told me she didn’t want me to come to the funeral if I was “going to be foul,” which I didn’t understand but was hurt by nonetheless … (P2957, female, 41)

The intended audience for this display—other family members, wider mourners, or just the mother—is unclear, but the daughter clearly felt pressure to cooperate with her mother in displaying a “happy family.” Having family unity displayed may be important not just to members of the deceased person’s family themselves but also to other mourners.

The main feeling I was aware of at the funeral was of some sense of a celebration of a life lived but a lack of heartfelt, vibrant, simple love and closeness. From talking to K and her daughter, we’d had the impression that there had not been a very close, loving bond but more one of duty and a wish to do the “right thing”… It was all a bit flat and unemotional, somehow. (H1745, female, 59, neighbor of deceased)

Thus we see that, whether or not writing as themselves ‘family’, correspondents shared notions of what a “good” family looks like at a funeral. First, the right members of that family should be present, whether or not they wanted to be there and whether or not they felt grief at the deceased’s demise; family members who absented themselves were liable to censure. Second, there should be a display of harmony and affection between the family members who are there.

### Feeling

Mourners shared expectations that family could be accomplished not only through attendance and seating but also through emotions and their expression. For Collins (2004), the stratifying function of rituals, separating insiders from outsiders, is built on emotion: “the experience of heightened mutual awareness and emotional arousal gives rise to group emblems, markers of group identity” (p. 36). Certainly in the M-O data, emotional display *and* inner feeling accomplish family membership. We now examine each in turn.

#### Display rules

In the following extract, a correspondent notices the different ways her boyfriend’s family and her own family are “done” at funerals:

It was my boyfriend’s granny’s funeral … After the Church service, we went to the cemetry in the car—this is the only time I saw b.f. mum cry and no one really spoke to each other at all … I just kept thinking that if this was my granny’s funeral then I would be very, very sad and that my mum would have been very involved—and that people would be talking to one another. (J4505, female, 28)

Another correspondent illustrates the “givenness” of family practices, explaining that even a funeral (she describes her aunt’s) is no reason to depart from how her family does emotional expression:

Then each row went to the coffin to pay their last respects—which I found very difficult. I couldn’t bear to look at the coffin or anyone else and just stared at the floor. I didn’t want to start openly really crying as no one else was. We’re not a family to show feelings of any sort. It would have been embarrassing to see me crying. (W3994, female, 38)

Emotional expression could also be a component of display, and once again, could be noticeable for its absence:

Both me and my husband were in tears, but what was strange was my husband’s sister looked quite relaxed and impassive. She showed no emotion at all. (R4365, female, 29, describing the funeral of her husband’s grandfather)

This writer is assessing how mourners display relatedness to the deceased through emotional expression. She compares her sister-in-law who was not crying with her own husband, who was, and it is the husband who is considered to be in line with the norm. What that norm is is not made explicit, but it seems safe to suggest that it has to do, as discussed earlier, with culturally held ideas about emotionally close relationships being at the heart of “proper,” “normal,” and “good” families.

Display rules vary from family to family and are one way that members do *our* family, but in the emotionally charged and publicly visible setting of the funeral this can be hard work, truly emotional *labor* (Hochschild, 1983), visible both to other members of one’s own family and to members of other families who may embrace different display rules.

I remember trying very hard to keep my emotions in check, as most of the family were doing so and it would have seemed very inappropriate not to follow suit. (R4695, male, 46)

Hockey’s (1993) interviews with clergy demonstrated their efforts to stage manage rules for emotional display—to control, and on occasion encourage, mourners’ emotions at funerals. But in the M-O data, the most commonly acknowledged guide to what is appropriate emotional behavior is neither funeral personnel nor general cultural rules, but the deceased’s family—though that can be problematic when the deceased’s and the correspondent’s family embrace incompatible display rules.

#### Feeling family

As well as mourners doing and displaying family through overt actions, they can also subjectively feel it, and these feelings can take center stage in correspondents’ writing:

I think the whole day was infected by the great sadness of a large family of 7 reduced to one old woman (me). When I was told over the phone that my brother had died, I suddenly felt lonely. This large, squabbling group of people you’ve known all your life has disappeared. My beloved family of course rally round + give me great comfort, + now + again you see aged faces—a schoolfriend, still recognisable, looms up! But being the last is salutary. (F1560, female, 89)

This correspondent describes what it feels like to lose family. What we want to draw attention to with the term “feeling family” is that at the funeral other mourners are conscious of this. They are conscious of the interiority of family relationships (Gabb, 2011) as well as external display. Correspondents considered the loss of family to hold a particular quality, with particular consequences, not usually shared by the bereavement of other relationships:

I felt sad for the adult children of the dead man who would miss him so much. (D826, female, 60)

She left a husband and two sons who were about 10 and 12 years old. It was unbelievably moving to see her sons there … It was the sadness of knowing that her sons would have to grow up without this fabulous woman. She adored her sons and so it was the pain of that which made it so hard. (J2891, female, 46)

I also felt very sad that the deceased’s mother should have to live to see her son die. (B4672, female, 29)

Correspondents—including those who were themselves members of the extended family—contrasted their own feelings of sorrow with the feelings of close family, according them more sympathy than they claimed for themselves:

… my aunt had played a significant part in my life—so I did feel sadness. And I felt particular sadness for my (widowed) 91-year-old uncle—who was having to cope with the end of his 64-year marriage. (C3603, male, 67)

When correspondents did sympathize with nonfamily, they also acknowledged the family’s loss:

The saddest funeral I can recall going to would be that of my cousin—she died aged 35 leaving three kids aged under 10. Now that was sad, not just from a personal and family point of view but seeing the misery on her friends faces. (S4429, male, 43)

Correspondents felt sorriest for the deceased’s coresident nuclear family. It is a simple point, but one that is crucial if we are fully to understand the authority that family holds for mourners at a funeral, since, as Morgan (1996) has noted, “any account of family that excludes emotions will be defective” (p. 123).

Unlike display rules which close family felt they had to conform to or whose variations from one family to another they observed, correspondents did not write about feeling family as though these feelings were subject to rules, still less that they had to do inner work in order to feel these feelings (Hochschild, 1983). Rather, their writing used the feelings evoked by the ritual setting to construct family; it is possible, of course, that they also verbalized this “feeling family” in informal social interaction at or after the funeral, though they do not state this.

Durkheim, Bloch, Davies (2002), and Collins have all written about the emotional energy produced in ritual, and how participants can use this to construct society. Certainly, correspondents who were not close family used this emotional energy to feel and construct family. Of course it is also possible, though we do not have evidence from the data, that some mourners—especially chief mourners—experience funerals as emotionally draining.

## Discussion

The trend, evident in scholarship and arguably also in society, is toward fluid, personalized understandings of both family and grief. Our empirical funeral data, however, contradict these trends: Our English correspondents privilege structural rather than fluid notions of both family and grief. As well as bringing together everyone who knew, cared for, and respected the deceased, these funerals differentiate family from nonfamily. All attenders, not just family members, actively contribute to the production and reproduction of cultural constructions of family. Every member of the funeral congregation is “in it together”—not in Durkheimian mechanical solidarity, but in collaboratively displaying and differentiating, through emotion, who is and who is not “family” in ways that, at least for M-O correspondents, are remarkably conventional. The funerals are portrayed as generating stratification more than solidarity, or perhaps more accurately, stratified solidarities. In the social order of these English funerals, this social differentiation is described as more important than displaying personal attachment to the deceased or intensity of grief; attempts to demonstrate attachment or detachment incommensurate with formal relationship were potentially problematic.

In making the distinctions that underlie their feelings, correspondents were drawing on a particular understanding of family, an understanding that did not appear to vary much, if at all. For correspondents, “family” in the context of funerals was constituted by a structured set of consanguinal and conjugal relationships, embodying structure more than fluidity (Edwards et al., 2012; Gilding, 2010). In other words, correspondents were drawing on a conventional—and normative (Reimers, 2011)—notion of family. Concepts that decenter conventional notions of family, such as families of choice (Weeks et al., 2001), “personal communities,” (Pahl & Spencer, 2004) “cultures of intimacy,” (Roseneil & Budgeon, 2004) or disenfranchised grief (Doka, 2002) did not feature in the data.

Why might this be? We can think of three possible explanations: methodological, demographic, and symbolic. First, the sample of correspondents was biased toward middle-class, White, older, females—the very people most likely to hold traditional notions of family and of grief. Because this is a qualitative project that does not claim its writers to be representative of the entire society, we note this limitation but feel no need to apologize for it. It is a significant enough finding that in the writings of M-O correspondents—reflecting a substantial element of English society—funerals are more about family than about grief, or at least that grief is mediated through conventional understandings of family.

Second, the “families of choice” concept was originally developed in the context of nonheterosexual people’s lives and relationships, which are not well represented in the M-O funeral data. Possibly there is a cohort effect; divorce rates did not rise significantly in Britain until the 1970s, while gay relationships were often secret until much later. Given that half of divorces occur within the first 10 years of marriage and given the elderly age of most deceased, it may take another decade or two until more diverse forms of personal life are fully represented in funerals.

Third, given the centrality of fertility symbols in many non-Western death rites (Bloch & Parry, 1982), we could speculate that M-O correspondents’ focus on a conventional understanding of family as the social unit of reproduction places the deceased within an ongoing process in which decay is countered by reproduction, with the deceased’s children and grandchildren potent symbols of new life that cannot be represented by friends or colleagues, however, long standing and close. If funerals are a rite symbolically conquering death (Davies, 2002; Bailey & Walter, 2016), then displaying biological family would be a prime way to do this. The M-O data neither support nor undermine this particular interpretation, though anecdotal stories we have heard of younger children being kept away from funerals do not support it.

The concept of disenfranchised grief would predict that a funeral’s focus on formal kinship would not only marginalize the grief of certain mourners and thus distress them but also suggest a failing of such funerals. Lifelong close friends excluded from “family-only” funerals certainly do appear in our data. These friends felt cheated, but no correspondent challenged the family’s right to exclude. Contrasting with the extensive literature on disenfranchised grief, they accepted the disenfranchisement; they accepted that funerals are ultimately about displaying family not displaying grief. Indeed, while some correspondents commented on a close family member’s grief being enfranchised even though little or no grief was actually present, no nonfamily attender bemoaned the funeral’s disenfranchisement of their own grief. Attenders’ writings construe the funeral as more to do with family than with personal attachment or its resultant grief.

It is possible that people generally, and particularly older middle-class English women raised in an era of stoicism (Jalland, 2010), find it easier to write about family than about grief, so that what they wrote about grief was mediated through writing about family. If so, our finding that grief at the funeral is mediated through conventional understandings of family may be a finding not about how people experience funerals but about how they retrospectively *write* about funerals. This limitation would apply also, of course, to interview material about experiencing funerals which, strictly speaking, would tell us only what was coconstructed in the interview rather than what was actually experienced at the funeral. Direct observation of funerals, by contrast, would inform us about behavior, but not about subjective meaning or how feeling rules are experienced. Each methodology has its limitations.

## Conclusion

How Western funerals accomplish family has been ignored in the research literature. This may reflect psychologists’ focus on grief rather than family, but even sociological and anthropological literature on Western funerals, which might be expected to focus on family and kinship, has not. Both scholarly and some popular writing has asserted that contemporary Western funerals are occasions for expressing grief and sharing sorrow; for celebrating the deceased’s unique life; or for bringing all the mourners together in an act of solidarity, either for their own sake or for the sake of supporting the family (Fulton, 1994). Correspondents’ writings, however, portray far more to “family” than offering support to family members. Walter’s (1994) analysis of death trends discussed the shifting authorities of tradition, religion, professional expertise, and the individual but ignored the authority of the family. By contrast, correspondents described funerals driven far more by conventional displays of family than by unbridled individualism.

Analyses of funerals as symbolizing group solidarity have failed to see the possibility that contemporary funerals may differentiate insiders from outsiders, stratifying the congregation into family and nonfamily, thus (re)producing rules as to what emotion may or may not be displayed. But this is what correspondents describe. They portray emotional display rules based on this dichotomy, with different rules for the members of each group; membership is surprisingly uncontested, while the classification of family is itself partly a product of emotions evoked by the ritual. In line with Randall Collins, and possibly against Douglas (2002), this classification is not simply cognitive but is accomplished through often deeply felt emotions. Displaying family at the funeral is thoroughly intertwined with displaying grief and feeling sorrow, with causal connections both ways: perceptions of “family” prompt emotions which in turn define family.

Correspondents accomplish family through what they write about grief, and they construct their own and others’ grief in terms of family connection to the deceased; in that sense, grief is trumped by family. That no nonfamily complained of their grief being marginalized suggests that our project correspondents are content, at least in the context of the funeral, with display rules that require grief to reflect family membership rather than personal attachment to the deceased. The notion, widespread in American thanatology (Doka, 2002), that disenfranchised grief is in all circumstances a bad thing to be challenged is not a view expressed by these largely older, middle-class English writers. They do not write of grief as an inalienable human right to be enfranchised in all circumstances, rather they write acceptingly of funerals they have attended where formal relationship to the deceased validates grief. It is of course possible that writers from other less stoical cultures might be less accepting of grief being disenfranchised. It is also possible that M-O correspondents might criticize grief’s disenfranchisement in other contexts than the funeral.

Strictly speaking, English funerals do not accomplish family; rather, M-O correspondents’ writings accomplish family. Were we to conduct interviews, the interview dialogue might accomplish family. Were we to observe funerals, we might interpret what we observe as the accomplishment of family. In each case, we may ask if it is the funeral, or the methodology, that accomplishes family. At the very least, this prompts the question whether other methodologies, such as direct observation or interviews (whether with mourners or with the funeral personnel who are skilled observers of mourners), would produce similar interpretations, or whether other methodologies would simply conclude that funerals deal in both grief and family, without one trumping the other, without grief *having* to be articulated through kinship connection. It also raises the question what might be found by researching other groups of British mourners or mourners in other Western societies.

## Notes

1 The concept of disenfranchised grief is sociological in that it recognises that bereaved people are subject to social norms, yet also a-social in that its ideal is an individualistic utopia in which every experience of grief is enfranchised through the abolition of social norms (Robson & Walter, 2012–2013).

2 See Bailey (2012, pp. 152–153), pp. 152–153, for reflections on the merits or otherwise of this exclusion.
